# Alpha 7 Nicotinic Acetylcholine Receptor Agonist PHA 568487 Reduces Acute Inflammation but Does Not Affect Cardiac Function or Myocardial Infarct Size in the Permanent Occlusion Model

**DOI:** 10.3390/ijms25084414

**Published:** 2024-04-17

**Authors:** Filip Mjörnstedt, Azra Miljanovic, Rebecka Wilhelmsson, Malin Levin, Maria E. Johansson

**Affiliations:** 1 Department of Physiology, Institute of Neuroscience and Physiology, The Sahlgrenska Academy, University of Gothenburg, 405 30 Gothenburg, Sweden; filip.mjornstedt@gu.se (F.M.); rebecka.wilhelmsson@gu.se (R.W.); 2 Wallenberg Laboratory, Department of Molecular and Clinical Medicine, Institute of Medicine, The Sahlgrenska Academy, University of Gothenburg, 413 45 Gothenburg, Sweden; azra.miljanovic@wlab.gu.se (A.M.); malin.levin@wlab.gu.se (M.L.)

**Keywords:** alpha 7 nicotinic acetylcholine receptor (α7nAChR), myocardial infarction, inflammation, α7nAChR agonist

## Abstract

Stimulation of the alpha 7 nicotinic acetylcholine receptor (α7nAChR) has shown beneficial effects in several acute inflammatory disease models. This study aims to examine whether treatment with the selective α7nAChR agonist PHA 568487 can dampen inflammation and thereby improve cardiac function after myocardial infarction in mice. The possible anti-inflammatory properties of α7nAChR agonist PHA 568487 were tested in vivo using the air pouch model and in a permanent occlusion model of acute myocardial infarction in mice. Hematologic parameters and cytokine levels were determined. Infarct size and cardiac function were assessed via echocardiography 24 h and one week after the infarction. Treatment with α7nAChR agonist PHA 568487 decreased 12 (CCL27, CXCL5, IL6, CXCL10, CXCL11, CXCL1, CCL2, MIP1a, MIP2, CXCL16, CXCL12 and CCL25) out of 33 cytokines in the air pouch model of acute inflammation. However, α7nAChR agonist PHA 568487 did not alter infarct size, ejection fraction, cardiac output or stroke volume at 24 h or at 7 days after the myocardial infarction compared with control mice. In conclusion, despite promising immunomodulatory effects in the acute inflammatory air pouch model, α7nAChR agonist PHA 568487 did not affect infarct size or cardiac function after a permanent occlusion model of acute myocardial infarction in mice. Consequently, this study does not strengthen the hypothesis that stimulation of the α7nAChR is a future treatment strategy for acute myocardial infarction when reperfusion is lacking. However, whether other agonists of the α7nAChR can have different effects remains to be investigated.

## 1. Introduction

The rupture of an atherosclerotic plaque and the subsequent formation of a thrombus in a coronary artery represents the major mechanism of acute myocardial infarction [1,2]. The thrombotic occlusion will cause the cardiomyocytes in the ischemic myocardium to die, which results in an immense inflammatory reaction [3]. A balanced inflammatory process is crucial for the repair and recovery of the myocardium, but excessive inflammation can damage the heart and contribute to heart failure. Therefore, novel pharmacological interventions that can target specific components of the inflammatory response has been suggested [4,5,6]. The cholinergic anti-inflammatory pathway describes a connection between the autonomic nervous system and the immune system. Experimental studies have shown that stimulation of the vagal nerve, the main component of the parasympathetic nervous system, can suppress the production of proinflammatory cytokines [7]. Hence, electrical and pharmacological stimulation mimicking vagal nerve signaling has been investigated in several inflammatory diseases such as atherosclerosis, myocardial infarction and rheumatoid arthritis [8,9]. The exact mechanism whereby stimulation of the vagal nerve exerts its immunomodulatory effects remains unknown, but accumulating data shows that stimulation leads to the release of acetylcholine that binds to the alpha 7 nicotinic receptor (α7nAChR). The α7nAChR is expressed on immune cells; hence, several specific agonists have been developed to investigate whether stimulation of the α7nAChR can have immunomodulatory effects [10,11]. Our group has recently shown that stimulation of α7nAChR with the agonist AZ6983 decreases atherosclerosis via immunomodulatory effects [12]. Two previous myocardial infarction studies have shown that treatment with PNU-120596 (positive allosteric modulator of the α7nAChR) and PNU-282927 (selective α7nAChR agonist) can decrease both the infarct size and the production of proinflammatory cytokines after myocardial infarction in rats using a reperfusion injury model [13,14]. In line with this, α7nAChR knockout mice have been shown to experience increased infarct size and impaired cardiac function after myocardial infarction [15]. However, whether stimulation of the α7nAChR with an agonist can affect the cardiac function following a myocardial infarction induced by permanent occlusion in mice remains to be investigated. Therefore, the aim of this study is to examine whether treatment with the selective α7nAChR agonist PHA 568487 [16] can dampen inflammation and thereby reduce infarct size and improve cardiac function following a myocardial infarction.

## 2. Results

### 2.1. α7nAChR Agonist PHA 568487 Dampens Inflammation in the Air Pouch Model

**Figure 1:**
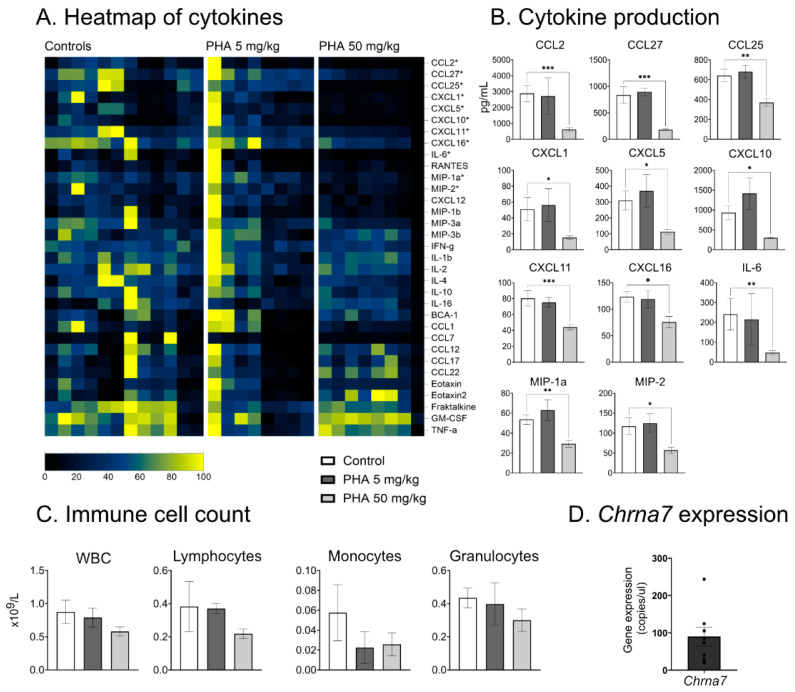
PHA 568487 decreases the concentration of pro-inflammatory cytokines in the air pouch model of inflammation. (**A**–**C**) Acute immunomodulatory properties of PHA 568487 were tested using the air pouch model of inflammation. Mice were treated with PHA 568487 12.4 μmol kg^−1^ (5 mg/kg) or PHA 568487 123.4 μmol kg^−1^ (50 mg/kg) i.p. and subsequently challenged with LPS into the pouch, *n* = 8–12 per group. Concentrations of cytokines in the air pouch exudate were analyzed after 6 h by multiplex assay and presented as a (**A**) heat map and (**B**) bar graphs representing cytokines that were significantly different compared to the controls. (**C**) Immune cell counts were measured with a Vetscan HM5 Hematology Analyzer. (**D**) Absolute quantification of *Chrna7* mRNA expression in primary mouse cardiomyocytes from adult mice (*n* = 7), using droplet digital PCR, gene expression presented as copies/µL. Kruskal–Wallis test was used, and data are expressed as mean ± SEM. * *p* < 0.05, ** *p* < 0.01, *** *p* < 0.001.

To determine the dose and possible anti-inflammatory effect of α7nAChR agonist PHA 568487, we used the acute inflammatory air pouch model [17]. In the air pouch model, treatment with high-dose PHA 568487 (50 mg/kg) significantly decreased the concentration of 12 cytokines compared to control mice (Figure 1A,B). After six hours of inflammation, IL6, CCL27, CXCL5, CXCL10 (IP-10), CXCL11, CXCL1 (GRO1), CCL2 (MCP-1), MIP1a, CXCL2 (MIP2), CXCL16, CXCL12 (SDF-1) and CCL25 were significantly decreased in the group receiving the high-dose PHA 568487. Treatment with low-dose PHA 568487 (5 mg/kg) did not affect the concentration of cytokines. There was no significant difference in the immune cell count of the air pouch exudate (Figure 1C).

### 2.2. α7nAChR Is Expressed in Primary Cardiomyocytes

To investigate a possible role for the α7nAChR in the heart, we first examined α7nAChR expression level in the heart. Using droplet digital PCR (ddPCR), gene expression of the gene coding for α7nAChR, *Chrna7*, in isolated primary cardiomyocytes was confirmed (Figure 1D).

### 2.3. No Effect of the α7nAChR Agonist PHA 568487 in the Permanent Occlusion Model of Myocardial Infarction

Next, we aimed to investigate whether the α7nAChR agonist PHA 568487 has a beneficial effect on cardiac function following myocardial infarction. The mean mortality rate of the control and PHA group was 37% (35% in the PHA group, 38% in the control group). There was no mortality in mice undergoing sham surgery.

**Figure 2:**
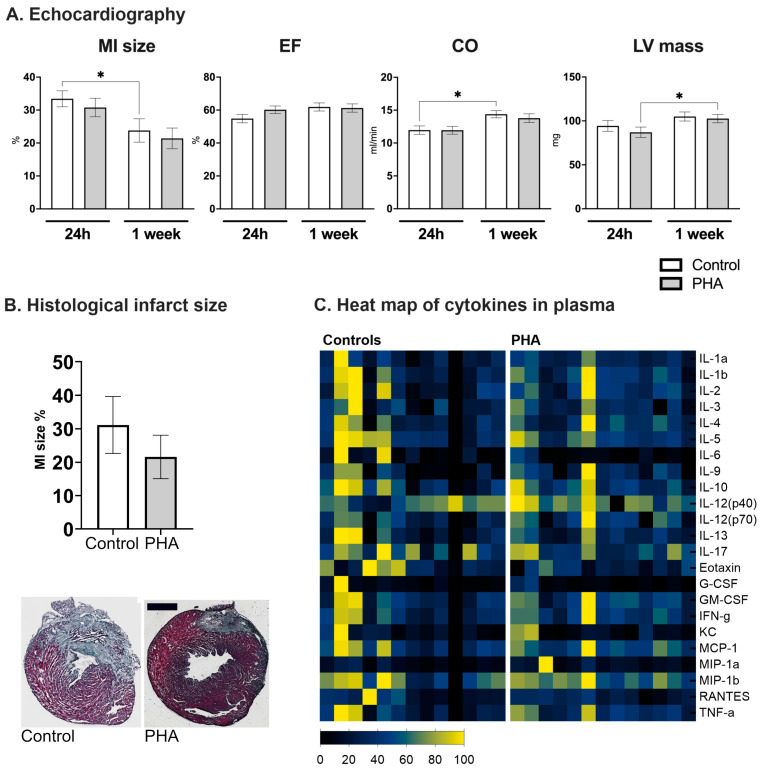
PHA 568487 did not affect the cardiac function, myocardial infarct size nor the cytokine levels in plasma. (**A**) Cardiac function and infarct size were assessed via echocardiography 24 h and one week after the myocardial infarction. (**B**) To verify the echocardiographic measurements, infarct size was also assessed histologically after sacrifice, one week after MI. Scalebar indicates 1000 µm. (**C**) Concentrations of 23 cytokines in plasma were analyzed at the day of sacrifice via multiplex assay. MI size: myocardial infarction size, EF: ejection fraction, CO: cardiac output, LV mass: left ventricular mass. In (**A**), Friedmans test was used followed by Dunn’s multiple comparison test. In (**B**,**C**), Mann–Whitney U test was used. Data are expressed as mean ± SEM. * *p* < 0.05, *n* = 13 per group.

Cardiac function was assessed using echocardiography at 24 h and 7 days post myocardial infarction. No difference was seen in either myocardial infarct size, ejection fraction, cardiac output or left ventricular mass when comparing mice treated with PHA 568487 (50 mg/kg) with saline-treated controls (Figure 2A,B). Furthermore, there was no difference between the groups in terms of heart rate, left ventricular volume, stroke volume, cardiac output, fractional shortening, fractional area change or left ventricular mass (Table 1). When investigating the cardiac parameters over time, from the 24 h time point to the 7-day time point, control mice recovered in terms of infarct area, as well as in cardiac output, an effect that was not seen in mice receiving PHA 568487 (Figure 2A). PHA568487-treated mice, on the other hand, demonstrated an increase in LV weight (Figure 2A) assessed by echocardiography. In sham mice, there was no significant difference in cardiac function 24 h post surgery (control vs. PHA, ejection fraction: 70 ± 6.6 vs. 60.3 ± 17.6, cardiac output 13.3 ± 1.3 vs. 12.7 ± 2.5; left ventricular mass 82.5 ± 6.6 vs. 99.3 ± 14.9, *n* = 3 in each group) nor at one week post sham surgery (control vs. PHA, ejection fraction 71.7 ± 7.5 vs. 58.4 ± 12, cardiac output 16 ± 3.5 vs. 12.6 ± 2.4; left ventricular mass 106.7 ± 25.7 vs. 103.8 ± 15.3, *n* = 3 in each group).

At the day of sacrifice, the white blood cell count (Table 1) and the concentration of 23 cytokines (Figure 2C) in the blood were analyzed; however, no differences were detected. Furthermore, there was no difference in sham mice receiving PHA 568487 compared to control mice receiving saline (Table 1).

## 3. Discussion

In this study, we found a clear anti-inflammatory effect of the α7nAChR agonist PHA 568487 in the air pouch model of acute inflammation. However, despite this clear anti-inflammatory effect in the acute model, treatment with the α7nAChR agonist PHA 568487 did not alter infarct size or cardiac function at 24 h or one week post myocardial infarction.

Recent studies have shown that treatment with the α7nAChR agonists GTS-21 and PNU-282927 can acutely decrease infarct size and the production of serum proinflammatory cytokines after myocardial infarction in rats [18,19], where histological assessments revealed a decreased infarct area in the rats treated with GTS-21 and PNU-282987 1.5 and 2.5 h post infarction, respectively. Importantly, in these two studies, the ischemia–reperfusion (I/R) model of myocardial infarction was used, whereas in the current study, we used a permanent occlusion model of myocardial infarction. The I/R method is considered to reflect the clinical setting of acute myocardial infarction, where restoration of blood flow plays a key role in the treatment strategy [20]. Importantly, permanent coronary occlusion is also a relevant animal model of acute myocardial infarction since it represents patients who, due to contraindications or logistic issues, do not receive timely or successful reperfusion [21]. Hence, both models contribute important information in different clinical settings. Nevertheless, taken together, these studies indicate that stimulation of the α7nAChR is beneficial following an ischemia–reperfusion injury, however, not as favorable in the permanent occlusion model.

The effect of α7nAChR stimulation on cardiac function after myocardial infarction has been less studied. In a model of ischemic cardiomyopathy, Lin et al. [22] reported cardioprotective effects when rats received daily treatment with α7nAChR agonist PNU-292987 for four weeks, demonstrating an improved ejection fraction in rats receiving α7nAChR agonist PNU-292827. Furthermore, mice lacking the α7nAChR display an increased infarct size and decreased ejection fraction four weeks post acute myocardial infarction compared with control mice [15]. These studies are in contrast with the current study, where we do not detect any difference in the functional parameters. Rather, control mice recover better, with decreased infarct size and improved CO one week after the myocardial infarction, an effect not seen in PHA 568487-treated mice. Additionally, PHA 568487-treated mice had increased left ventricular mass, indicating worse performance compared to control mice. The discrepancy between these findings needs further evaluation but could potentially be due to the different models used as well as the different treatment strategies, both in terms of agonist used as well as number of treatments.

The air pouch model is a broadly used model of acute inflammation to identify potential anti-inflammatory drugs in inflammatory diseases [17,23,24,25]. Using the air pouch model, we determined the dose of α7nAChR agonist PHA 568487. Interestingly, out of the 12 cytokines significantly decreased by the α7nAChR agonist PHA 568487, besides from IL-6, all of the other cytokines are involved in recruitment, i.e., acting chemotactic for neutrophils (CXCL1, CXCL2) [26], monocytes (CXCL10, CCL2, CCL25) as well as for T cells (CCL27, CXCL10) [27]. Despite the dampening effect of α7nAChR agonist PHA 568487 on several different chemokines, this did not alter the number of recruited cells to the air pouch.

Although, α7nAChR agonist PHA 568487 suppressed cytokine levels in the air pouch model, no differences were found in the white blood cell count or in the concentrations of cytokines and chemokines when analyzing the blood from the day of sacrifice. Possibly, the suppression of cytokines by PHA 568487 is local, as seen in the air pouch experiments, and not a systemic effect detected in blood. Another possible explanation is that the sacrifice took place six days after the final injection of PHA 568487 and that the blood concentration of immune cells and cytokines could have time to go back to normal during this time. A more intense treatment protocol would perhaps show a different result. Another limitation of the current study is that we give the first treatment before the induction of myocardial infarction, which would be hard to mimic in a clinical setting.

## 4. Materials and Methods

### 4.1. Isolation of Primary Cardiomyocytes and Droplet Digital PCR (ddPCR) Analysis

Isolation of primary cardiomyocytes from C57Bl/6N male mice (Taconic, Borup, Denmark) was carried out as previously described [28]. RNA from cardiomyocytes (*n* = 7) was extracted using the RNeasy Mini Kit (Qiagen GmbH, Hilden, Germany) according to the manufacturer’s protocol. Reverse transcription was performed using the High-capacity cDNA Reverse Transcription Kit (Applied Biosystems, Waltham, MA, USA), according to the manufacturer’s protocol. The expression of the α7nAChR gene, *Chrna7*, was determined with droplet digital PCR (ddPCR) utilizing EvaGreen (Bio-Rad Laboratories, Hercules, CA, USA) as previously described [29]. Samples were analyzed in the QX200 Droplet Reader (Bio-Rad Laboratories) and the results, including positive and negative droplets, analyzed with QuantaSoft Analysis Pro™ (Bio-Rad Laboratories).

### 4.2. Air Pouch Model

To evaluate the possible immunomodulatory effects of PHA 568487 in vivo, the air pouch model of acute inflammation was used [12,25]. At 8 weeks of age, male C57BL/6JRj mice (Janvier Labs, Le Genest-Saint-Isle, France) were injected with 3 mL of sterile air between the scapulae under isoflurane anesthesia. Three days later, the air pouch was re-filled with 3 mL of sterile air. On the sixth day, the mice received an intraperitoneal injection of either PBS (Thermo Fisher Scientific, Waltham, MA, USA) or PHA 568487 (Tocris Bioscience, Ellisville, MO, USA) at 12.4 μmol kg^−1^ (5 mg/kg) or 123.4 μmol kg^−1^ (50 mg/kg). Fifteen minutes later, all mice were injected with LPS (10 μg, List Biological Laboratories Inc., Campbell, CA, USA) directly into the pouch. The mice were sacrificed six hours after the LPS injection by an overdose of pentobarbital (Apoteket AB, Stockholm, Sweden). Finally, the pouch cavity was flushed with 2 mL of PBS with 1.6 mmol L^−1^ of EDTA, and the exudate was collected. The white blood cell count of the exudate was analyzed using the Vetscan HM5 Hematology Analyzer (Abaxis, Union City, CA, USA) and thereafter centrifuged for 5 min at 400× *g*. Supernatants were stored at −80 °C until multiplex analysis.

### 4.3. Myocardial Infarction Study

A total of 47 mice were included in the myocardial infarction study, performed at two separate time points. Twelve-week-old male C57BL/6JRj mice (*n* = 47) were purchased from Taconic (Denmark) and randomly assigned into two groups: Control (receiving PBS, *n* = 21) and PHA (receiving PHA 568487, *n* = 20). A permanent left anterior descending artery (LAD) occlusion was performed as previously described [30]. Each treatment group had a corresponding sham group (*n* = 3 + 3) undergoing the same surgical protocol with the exception of LAD ligation. Five minutes before the surgery, the mice received an intraperitoneal injection of either PBS (control group) or PHA 568487 (123.4 μmol kg^−1^, 50 mg/kg). The chest of each mouse was shaved. Further, the mice were anesthetized, orally intubated and connected to a small animal ventilator distributing a mixture of oxygen and 2–3% isoflurane. The mice were placed on a heating pad (Rodent Surgical Monitor, Scintica, London, ON, Canada) where ECG was continuously measured. A rectal probe measuring temperature was applied, and the core temperature was maintained between 36.5 and 37.5 degrees Celsius. An incision was made between the 4th and 5th ribs to reveal the left ventricle. The pericardium was opened, and myocardial infarction was induced by ligation of the left anterior descending coronary artery immediately after the bifurcation. Successful ligation was confirmed by characteristic ECG changes and akinesia of the ventricular wall. The lungs were hyperinflated, positive end expiratory pressure was applied, and the chest was closed. The mice received an intraperitoneal injection of Temgesic (0.1 mg/kg) to relieve postoperative pain and were allowed to recover spontaneously after turning off the isoflurane administration. The mice that did not survive the surgery all died within 10 min of chest closure. Twenty-four hours after the myocardial infarction, the mice received a second intraperitoneal injection of either PBS or PHA 568487 (123.4 μmol kg^−1^, 50 mg/kg). Mice were sacrificed 7 days after the myocardial infarction by an overdose of isoflurane. Heart tissue was perfused with saline, subsequently cooled in isopentane, snap-frozen in liquid nitrogen and stored at −80 °C until analysis. Blood was collected and analyzed using the Vetscan HM5 Hematology Analyzer (Abaxis, Union City, CA, USA) and thereafter centrifuged for 5 min at 400× *g*. Supernatants were stored at −80 °C until multiplex analysis.

### 4.4. Echocardiographic Measurements in Mice

Mice were anesthetized with isoflurane (1.2%) and underwent echocardiographic examination at two different time points: 24 h and 7 days after the myocardial infarction. Echocardiography was performed using VEVO 2100 (VisualSonics Ontario, Toronto, ON, Canada), including an integrated rail system for consistent positioning of the ultrasound probe as previously described [30]. In brief: The chest of each mouse was shaved, the mice were placed on a heating pad, and their paws connected to electrocardiographic (ECG) electrodes. A 45 MHz linear transducer (RMV 704) was used. An optimal parasternal long-axis cine loop was acquired to image the mitral and aortic valves and the maximum distance between the aortic valve and the cardiac apex. Parasternal short-axis cine loops were acquired at 1, 3 and 5 mm below the mitral annulus. End-diastolic and end-systolic left ventricular volumes and ejection fraction were calculated with the biplane Simpson’s formula using the 3 parasternal short-axis views and the parasternal long-axis view. For M-mode measurements (at the 3 mm level), the leading-edge method was used. End diastole was defined at the onset of the QRS complex, and end systole was defined as the time of peak inward motion of the interventricular septum. Infarct size was assessed based on wall motion score index (WMSI) [31] 24 h and 1 week after myocardial infarction by a 16-segment model on 3 short-axis images, as 0 for normal, 0.5 for reduced wall thickening and excursion in a segment and 1 for no wall thickening and excursion in a segment. WMSI was calculated as the sum of scores divided by the total number of segments. The stored data were evaluated offline in a blinded fashion with Vevo Lab software (VisualSonics).

### 4.5. Histological Analysis of Myocardial Infarction Size

Frozen hearts were embedded in OCT and cryosectioned in 10 µm sections. Sectioning begun from the apex, and sections were collected every 100 µm up until 700 µm. Sections were stained with a Masson–Goldner staining kit followed by Weigerts hematoxylin (both from Sigma-Aldrich, St. Louis, MO, USA, Merck KgaA, Darmstadt, Germany), according to the manufacturers protocol. The infarction area was calculated as the percentage of cardiac tissue for each level and presented as the average infarction area per heart.

### 4.6. Multiplex Analysis

The Bio-Plex Pro^TM^ Mouse Chemokine Panel 33-plex (#12002231, Bio-Rad Laboratories, Inc.,) and the Bio-Plex Pro Mouse Cytokine 23-plex Assay (#M60009RDPD, Bio-Rad Laboratories) was used to measure cytokines in the exudate from the air pouch model and serum cytokine levels in mice from the myocardial infarction study, respectively. The assays were performed according to the manufacturers’ protocol as previously described [12].

### 4.7. Viability

The viability of PHA568487-stimulated PBMCs was determined using alamarBlue Cell Viability Reagent (Invitrogen, Waltham, MA, USA), according to the manufacturer’s protocol. In brief, HL-1 cardiomyocyte cell line, a kind gift from Prof. W. Claycomb [32], plated in triplicates at a concentration of 20,000 cells/well, were incubated with or without PHA 568487 (1 µM, 10 µM or 100 µM) for 20–24 h prior to the viability test. Cells were subsequently incubated with alamarBlue Cell Viability Reagent for 4 h at 37 °C and the absorbance read at 570 nm, with 600 nm set as a reference wavelength. A viability test was performed in three independent experiments. There was no effect on viability of any of the PHA568487 concentrations.

### 4.8. Ethical Considerations

All procedures involving mice were approved by the Regional Animal Ethics Committee at the University of Gothenburg, in accordance with the European Communities Council Directives of 22 September 2010 (2010/63/EU).

### 4.9. Statistics

Data were tested for normality with Shapiro–Wilk’s test and statistical methods chosen according to the outcome. The data from the air pouch experiment, where the control group and two different concentrations of PHA 568487 were compared, were analyzed with the Kruskal–Wallis test followed by Holm–Sidak’s multiple comparison test. The data from the myocardial infarction study (echocardiographic measurements, multiplex assays, white blood cell count) were analyzed with Friedmans test followed by Dunn’s multiple comparison test when comparing 24 h and 1 week and with the Mann–Whitney U test when comparing controls with the PHA group. Data are expressed as mean ± SEM unless otherwise stated, and *p* < 0.05 was considered statistically significant.

## 5. Conclusions

In summary, treatment with the α7nAChR agonist PHA 568487 did not affect infarct size or cardiac function after a permanent occlusion model of acute myocardial infarction in mice. Consequently, this study does not strengthen the hypothesis that stimulation of the α7nAChR is a promising future treatment strategy for acute myocardial infarction when reperfusion is lacking. Possibly, targeting the α7nAChR in ischemia–reperfusion injury might have beneficial effects suggested by the previous acute studies; however, whether it also has an effect in a more long-term study needs to be further evaluated. In addition, we cannot rule out that other agonists of the α7nAChR can have different effects; hence, this remains to be investigated.

## Figures and Tables

**Table 1:** Cardiac and hematological parameters 24 h and 7 days post MI.

	24 h Post MI					One Week Post MI				
	Control	PHA	Sham Control	Sham PHA	*p*	Control	PHA	Sham Control	Sham PHA	*p*
n	13	13	3	3		13	13	3	3	
BW, g	24.2 ± 2.5	23.5 ± 2.5	23.8 ± 2.2	21.2 ± 1.9	0.52	25.1 ± 2.7	24.6 ± 2.6	24.9 ± 2.2	23.7 ± 2.8	0.65
HR, bpm	437 ± 55	440 ± 50	453 ± 24	394 ± 79	0.92	435 ± 51	421 ± 39	464 ± 54	365 ± 49	0.51
SV, μL	27.2 ± 4.6	27.1 ± 4	29.1 ± 2.1	31.9 ± 1.9	>0.99	33.4 ± 4.4	33.5 ± 5.4	34.3 ± 4	33.4 ± 8.9	0.92
FS, %	8.4 ± 4.8	10.8 ± 3.9	18.4 ± 5.9	10.7 ± 5.6	0.22	11.7 ± 6	11.5 ± 4.2	16.9 ± 7.3	13.1 ± 7.1	0.96
FAC, %	50.6 ± 8.2	55.9 ± 11.2	66.8 ± 6.3	53.9 ± 22.3	0.08	55.7 ± 9.8	56.4 ± 11.8	69 ± 8.5	52.1 ± 10.3	0.92
LV V(d), μL	50.3 ± 8.1	45.6 ± 8.7	41.8 ± 1.1	55.1 ± 11.2	0.13	55.0 ± 10	56.1 ± 12.7	47.8 ± 0.5	57.1 ± 8.1	0.92
LV V(s), μL	23.1 ± 7.7	18.5 ± 7.2	12.6 ± 3.1	23.2 ± 13.2	0.06	21.6 ± 8.7	22.5 ± 9.6	13.5 ± 3.5	23.8 ± 7.9	0.80
WBC, ×10^9^/L	NA	NA	NA	NA		3.7 ± 1.3	3.2 ± 1.1	5.1 ± 0.6	4.3 ± 0.1	0.38
Lym, ×10^9^/L	NA	NA	NA	NA		1.6 ± 0.7	1.8 ± 0.5	3.3 ± 0.8	2.6 ± 0.2	0.58
Gran, ×10^9^/L	NA	NA	NA	NA		1.9 ± 1.3	1.3 ± 1	1.7 ± 0.5	1.4 ± 0.3	0.19
Mon, ×10^9^/L	NA	NA	NA	NA		0.2 ± 0.2	0.2 ± 0.1	0.2 ± 0.2	0.3 ± 0.04	0.18

Echocardiographic measurements and immune cell populations. Treatment with PHA 568487 did not affect echocardiographic measurements of the heart 24 h and 7 days after myocardial infarction. No difference in immune cell population in blood was observed at the day of sacrifice. Body weight (BW), heart rate (HR), stroke volume (SV), fractional shortening (FS), fractional area change (FAC), left ventricular volume in diastole (LV V(d)), left ventricular volume in systole (LV V(s)), white blood cell count (WBC), lymphocyte count (Lym), granulocyte count (Gran), monocyte count (Mon). Mann–Whitney U test was used to compare the control and PHA group at the two different time points. Values are represented as mean ± SEM.

## Data Availability

Data are contained within the article.
